# Handwashing Knowledge, Attitudes, and Practices among Students in Eastern Province Schools, Saudi Arabia

**DOI:** 10.1155/2021/6638443

**Published:** 2021-09-21

**Authors:** Munthir M. Almoslem, Talal A. Alshehri, Arwa A. Althumairi, Mohammed T. Aljassim, Mohamed E. Hassan, Mahmoud M. Berekaa

**Affiliations:** ^1^Environmental Health Dept., College of Public Health, Imam Abdulrahman Bin Faisal University, P.O. Box 1982, Dammam 31441, Saudi Arabia; ^2^Health Information Management and Technology Dept., College of Public Health, Imam Abdulrahman Bin Faisal University, P.O. Box 1982, Dammam 31441, Saudi Arabia; ^3^Public Health Dept., College of Public Health, Imam Abdulrahman Bin Faisal University, P.O. Box 1982, Dammam 31441, Saudi Arabia

## Abstract

*Background*. Lack of knowledge about appropriate handwashing practices has caused great concerns for human health, especially in the risk of many communicable diseases. The objective of the current study is to determine the level of handwashing knowledge, attitudes, and practices among school students in Eastern Province Schools, Saudi Arabia. A cross-sectional survey was recruited from November 2019 to March 2020 to assess the level of the students' handwashing knowledge. A reliable questionnaire was prepared (Cronbach's alpha = 0.608) and conducted using a two-stage sampling technique. A total of 271 students participated in the study from primary, middle, and high schools; 80% were boys, most of whom displayed an acceptable level of knowledge on hand hygiene. Nearly 75% and 74% of boys and girls, respectively, gained knowledge about hand hygiene practices from their parents. Only 46% of the students thought that handwashing is a potential protective measure against diseases, whereas 34% thought it only removes dirt. Prevalence of handwashing with soap after using the toilet was recognized among 52% of the students. Additionally, 93% of the students used water and soap to wash their hands (*p* value < 0.001) and 97% suggested that soap and water are the best methods to wash their hands (*p* value < 0.001). There was a positive correlation between the mother's education and hand hygiene practices (*p* value = 0.044). Results collectively indicated that handwashing knowledge and practices among school students in the Eastern Province are acceptable interventions in preventing the transmission of infectious diseases such as COVID-19. Indeed, further improvement conducted through specific health education programs to emphasize the role of handwashing in health hygiene is highly recommended.

## 1. Introduction

Practicing appropriate handwashing with optimum frequency is a fundamental skill for leading a healthy life [1, 2]. Handwashing, especially before eating, is believed to be one of the first techniques to protect children, teens, and adults from many communicable diseases [1, 3]. Generally, there is a progressive increase in risks associated with a wide range of diseases directly correlated with handwashing, for example, water- and foodborne diseases, contagious diseases, severe acute respiratory syndrome (SARS), H1N1 influenza A, norovirus, cholera, malaria, dysentery, meningitis, shigellosis, and multiresistant *Staphylococcus aureus* [4]. In fact, childhood diarrhoea was found to be significantly correlated with handwashing without soap [5]. Generally, contaminated hands can be a source of infectious diseases, and this happens after picking one's nose or coughing, using the bathroom, and dealing with garbage [6]. Furthermore, handwashing is an essential cause for healthy growth and development in the community [2, 7, 8]. Unfortunately, handwashing after visiting the restroom is ill-practiced in many societies, notwithstanding its significant effect on human health [9]. Likewise, nonroutine handwashing is recorded as a major risk factor associated with head, foot, and mouth diseases (HFMD) among children in China and other Asian countries [10]. Generally, hospital-acquired infections can be decreased by the very simple but crucial intervention of handwashing [11]. Similarly, handwashing is considered an efficient preventive measure for children, with a subsequent reduction in child antibiotic use [12].

On the other hand, schools are one of the most important places for promoting health education and programs [3, 13, 14]. The students can gain knowledge, skills, and positive behaviours in terms of handwashing and many other hygiene practices [3, 13]. According to Al-Bashtawy [3], many students in developing countries have shown a lack of handwashing skills. With handwashing, a simple and largely cost-effective hand hygiene technique, many schools encourage their students to practice handwashing behaviours [3, 13].

Besides teachers, parents, and classmates' attitudes significantly influence handwashing and hygiene behaviours and habits among students [15–17]. Nevertheless, students' hygiene knowledge, attitudes, and practices have shown significant discrepancies between genders [18]. It has been found that the interventions of handwashing and personal hygiene in school children have led to a significant reduction in diarrhoea cases and absence rates among students [19]. Moreover, the intervention of handwashing has significantly improved schoolchildren's knowledge and practices, helping them to communicate the latter with their parents efficiently [20].

Several studies have been conducted to investigate the issues regarding handwashing and general hygiene in school students. Handwashing, especially after visiting bathrooms, has a significant effect on the spread of parasitic infections, with increased cases of the latter present among school students in many countries [13, 21, 22].

In Saudi Arabia, several studies have been conducted to explore students' knowledge and attitudes towards hand hygiene practices in many cities including Abha, Majma'ah, and Al-Ahsa [23–29]. On the other hand, no study has been conducted to assess handwashing practices and knowledge among school students in Eastern Province, Saudi Arabia. However, many researchers have studied general hygiene practices and food hygiene at a school level.

To fill the gap of knowledge on handwashing practices among school students in Eastern Province, Saudi Arabia, the current study was conducted for the first time. This study aims to determine the level of the school students' handwashing knowledge and practices in three different cities in Eastern Province (Dammam, Dhahran, and Khobar) and identify the sources of knowledge about hand hygiene. Furthermore, the study attempts to find out the correlation between student characteristics and hand hygiene practices.

## 2. Materials and Methods

### 2.1. Study Location

The study was conducted in selected schools in the major three cities in the Eastern Province (Dammam, Dhahran, and Khobar), Saudi Arabia, between November 2019 and March 2020.

### 2.2. Sampling Design

A cross-sectional survey was recruited from November 2019 to March 2020 to assess the level of the students' handwashing knowledge. Data collection was based on a survey questionnaire adapted from Dajaan et al. (2018). The questionnaire was translated to Arabic, and its contents were validated by public health experts. Cronbach's alpha test was performed using SPSS version 19.0 to check the reliability of the questionnaire and was found to be 0.608.

Random sampling was performed using a two-stage sampling technique that involved a fixed number of schools for each stratum in three major cities in Eastern Province, namely Dammam, Khobar, and Dhahran. In the first stage, every 35^th^ school was randomly selected from a list of 1,068 schools in the major three cities in the Eastern Province. In the second stage, every 3^rd^ student was randomly selected from a students' list of each school. The number of students spread proportionally across the schools, and 271 students were selected from 30 schools. In addition, a checklist was created to inspect the availability of handwashing facilities at schools. Assistance has been provided to respondents to explain the questions as well as to fill out the questionnaires. The questionnaires were collected on the same day of each visited school after assuring that all questions were answered with care and attention.

The questionnaire included questions on student's parental education and sociodemographic characteristics. In fact, there were 16 questions assessing the perceived levels of knowledge on hand hygiene practices, with a clear response to hand hygiene acquisition and performance. Indeed, the distribution of student responses on their hand hygiene practice was targeted by 11 questions.

### 2.3. Inclusion and Exclusion Criteria

Primary, intermediate, and secondary school students (male and female) from the selected schools were included in the study. However, students with mental or physical disabilities from the selected schools were excluded.

### 2.4. Statistical Analysis

The normality of data was tested using a boxplot diagram. A descriptive analysis was the first section presenting the percentage of responses to each domain. The bivariate analysis comprised a Chi-square score analysis for categorical two-group data and ANOVA for categorical data with more than two groups to assess the relationship between student characteristics and the score of hand hygiene practices. All statistical analyses were performed using the SPSS 19.0 program (SPSS Inc., Chicago, IL, USA).

### 2.5. Ethical Considerations

Ethical permission was obtained from the Institutional Review Board (IRB) of Imam Abdulrahman Bin Faisal University, Dammam, KSA (ethical permission number: IRB-2021-057-CPH).

## 3. Results

### 3.1. Students' Characteristics

Among the 271 students who participated in the study, 80% were boys, and 71% were above 14 years old, that is, mostly from high school (Table 1). There was great variation in terms of parental education, with half of the mothers holding high school and undergraduate degrees (26% and 35%, respectively), while two-thirds of the fathers had gained undergraduate and postgraduate degrees (27% and 28%, respectively).

**Figure 1:**
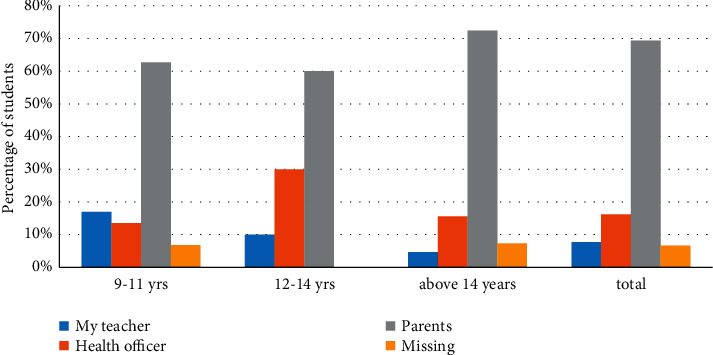
Major sources of knowledge about hand hygiene among school students between 9 and 14 years old.

Seventy-five percent of boys and 74% of girls agreed that they had learnt about hand hygiene practices from their parents (Table 2 and Figure 1). For all age groups, parents had the greatest impact as a means of transmitting handwashing education (Table 2).

As graphically presented in Figure 1, approximately 74–75% of students of all ages gained their knowledge of hand hygiene practices from their parents.

### 3.2. Perceived Knowledge of Hand Hygiene

Assessing the students' perceived knowledge on hand hygiene indicated that a majority of participants had a high level of knowledge on hand hygiene (Table 3). Moreover, most students (87%; *p* value = 0.001) recognized that washing hands with water and soap at schools is significant. Interestingly, 91.1% (*p* value = 0.001) of students had been educated on how to wash their hands. Unfortunately, only 46% thought that handwashing prevents diseases, and approximately 40% thought it removes dirt, whereas 69% did not believe that handwashing could remove germs. Additionally, 82.7% of students recognized the significant impact of handwashing on personal hygiene (*p* value = 0.001). Regarding the use of soap in handwashing, 77.5% of students washed their hands before and after eating, whereas 52% washed their hands after using the toilet and 83% while preparing food (*p* value = 0.001).

**Figure 2:**
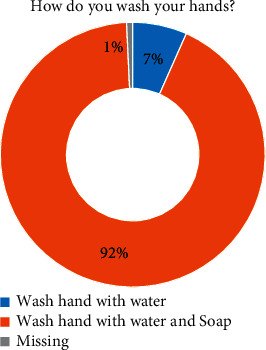
Student responses on their hand hygiene methods.

**Figure 3:**
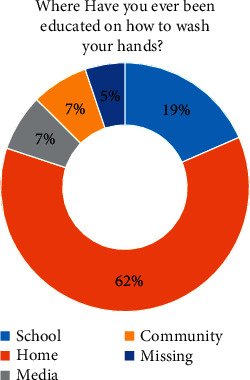
Student responses on the place of hand hygiene knowledge acquisition.

Regarding students' performance, 92% used water and soap to wash their hands (Figure 2), and 97% (*p* value < 0.001) agreed that using soap and water is the best method to wash their hands (Table 4). Surprisingly, 62% learnt to wash their hands at home and 18% at school (Figure 3).

The students were asked about their perceived hand hygiene practices (Table 5), with the majority of their answers showing evidence of positive practices and with more than 80% agreeing on the best forms of hand hygiene practice (*p* value < 0.001). However, when they were faced with confirming the statement “I always wash my hands after playing with friends,” only 61% did so.

A bivariate analysis to assess the relation between student characteristics and scores of hand hygiene practice (score of 12) was conducted. The analysis indicated that the student's gender and their father's level of education have no significant impact on perceived practices; however, age had a significant impact on their perceived practice (*p* value < 0.015), and the older the student, the more aware of hand hygiene practices they were. Additionally, the students' mothers' levels of education had a significant relationship to hand hygiene practices (*p* value = 0.044), as students with a mother who had an elementary or high school education (*p* value = 0.071 and 0.014, respectively) displayed the highest scores of perceived practices of hand hygiene (Tables 6 and 7).

## 4. Discussion

The current study was conducted to assess the level of handwashing knowledge and practice among students in the Eastern Province, Saudi Arabia. Consideration was given to the relationship between students' backgrounds and hand hygiene practices. Results revealed that most of the students (80%) possessed an awareness of hand hygiene. Similar findings were recorded among male primary school students in the city of Abha, Saudi Arabia [30]. In concordance, a study in Abha revealed that 86.6% of the students have acknowledged that respiratory tract infections can be reduced if the proper handwashing practices are maintained [26]. Moreover, Hazazi et al. [30] found that approximately 95% of the students realized the importance of hand hygiene, especially in disease spreading through person-to-person contact. Also, in a study to detect the level of knowledge and practice as a preventive measure to combat COVID-19 disease in Saudi Arabia, Siddiqui et al. [31] revealed that 84% of the population realizes and practices handwashing. On the contrary, Dajaan [15] revealed that only 37.67% of primary school students in Ghana realized the importance of handwashing in disease prevention. Compared to the studies carried out in other Saudi Arabia cities, Ghana, and India, school students in Eastern Province recorded a better awareness towards hand-hygiene-related knowledge and practices [8, 15, 32].

Approximately 75% of the students had learnt hand hygiene practices from their parents; these findings were expected, as the students in the study location generally come from a high socioeconomic status. On the contrary, Al-Hazmi et al. [25] revealed that knowledge of preventive measures against infectious diseases is higher among college students than in schools, and the media is the major source of information on those measures, rather than academic institutions' programs. Moreover, a study in Al-Ahsa region, Saudi Arabia, explicits that sociodemographic factors and personal hygiene habits are associated with the prevalence of infectious diseases [29]. Most of the students (97%) agreed that soap and water is the best method for washing their hands; this result was consistent with some other previous studies [13, 30, 33–35].

Only 46% of the students thought that handwashing prevents diseases, and 34% of them thought that it removes dirt. Concurrently, Dajaan et al. [15] have found that 100% of Saudi school students recognize the importance of soap and water in handwashing, with their results also showing that 37.67% of the respondents in the study washed their hands to prevent disease, and 21.33% washed their hands to remove germs and dirt [15].

Moreover, 86% and 87% of the students washed their hands before eating and after using the toilet, respectively. In Majma'ah, a study compared food hygiene practices, including handwashing, between primary, intermediate, and secondary school students and found that the students demonstrated good levels of practice, even though attitudes and levels of knowledge were considerably reasonable [28]. Hazazi et al. [30] revealed that more than 90% of primary year school students use soap in handwashing especially before and after eating and also after using the toilet. Unfortunately, only 39.88% of primary school students in Ghana use soap in handwashing after visiting the toilet [15]. Remarkably, UNICEF [36] declares that the two most vital moments of handwashing happen before eating and after using the toilet, supporting the findings of the current study.

Also, the results remarkably revealed that approximately 86% and 87% of the students washed their hands before eating and after using the toilet. The role of handwashing as an efficient preventative technique against many infectious diseases, for example, impetigo, diarrhoea, HFMD, and the novel COVID-19 has been reported by many scientists [4, 10, 35, 37, 38]. Recently, the role of hand hygiene behaviour in COVID-19 prevention among primary school students has been studied in China [39]. Approximately, 42.05% of the students showed good behaviour and were significantly affected by many factors including mother's educational background. In a study to detect the level of knowledge and practices during the COVID-19 pandemic among the Saudi population, it was found that most of the participants preferred handwashing to alcohol disinfection [40].

Indeed, the use of soap in handwashing by approximately 86.5% of the students is greater in comparison with other countries (42% to 49%) [4]. Also, handwashing with soap plays a crucial role in the prevention of water- and foodborne diseases by 50% to 70%, and pneumonia, impetigo, and diarrhoeal diseases by 40% to 50% [4]. Also, in Riyadh, a study revealed that handwashing with soap is negatively correlated with students' absence in school [27]. Interestingly, the prevalent use of soap with handwashing is recorded as an efficient measure for reducing contamination of the hands due to the potentially lethal effects of microbial contaminants [41, 42].

Finally, in concordance with previous studies, maternal education levels and student age are significantly correlated with good hand hygiene practices among school students. However, the gender of school students was not correlated with the level of handwashing. On the contrary, Alshammary et al. [43] revealed that Saudi females are equipped with a higher level of knowledge and practice on hand hygiene than males during the COVID-19 pandemic (86% and 80%, respectively). Moreover, Dajaan et al. [15] identified that female students have higher handwashing practices than males.

## 5. Conclusions

The purpose of the current study was to determine the level of school students' handwashing knowledge and practices, with a special emphasis on their source of knowledge about hand hygiene. Additionally, the correlation between students' backgrounds and hand hygiene practices was clearly discussed. Interestingly, more than 80% of the students have high levels of hand hygiene awareness, especially learnt at home. Unlike the students' fathers' education levels, their mothers' education levels, and the age of the students have been found to have a significant impact on hand hygiene practices and behaviour. The work presented in this study can serve as a basis for the construction of awareness programs to promote hand hygiene education and practices against infectious diseases, for example, COVID-19 and similar pandemics. Further studies are needed to understand how hand hygiene is practiced at home and in school while relating this practice to infectious disease transmission and risk factors.

## Figures and Tables

**Table 1:** Distribution of student characteristics.

Student characteristics	*N*	Percentage	Chi^2^ (df)	*p* value
	271			

Gender				
Boy	218	80	100.5 (1)	<0.001
Girl	53	20		
Age				
9–11 years	59	22	180.1 (2)	<0.001
12–14 years	20	7		
Above 14 years	192	71		
Class level				
Elementary school	59	22	180.1 (2)	<0.001
Secondary school	20	7		
High school	192	71		
Maternal education level			133.1 (5)	<0.001
No formal education	21	8		
Elementary and secondary level	23	9		
High school level	69	26		
Undergraduate level (diploma or bachelor's)	95	35		
Postgraduate level (master's, doctoral, or above)	37	14		
I do not know	26	1		
Paternal education level			121.9 (5)	<0.001
No formal education	15	6		
Elementary and secondary level	14	5		
High school level	61	23		
Undergraduate level (diploma or bachelor's)	74	27		
Postgraduate level (master, doctoral, or above)	76	28		
I do not know	31	1		

**Table 2:** The relationship between gender, age distributions, and the teaching means.

Student characteristics	Teacher		Health officer		Parent		Missing		Chi^2^ (df)	*p* value
	*n*	%	*n*	%	*n*	%	*n*	%		

Gender										
Boys	18	9	34	17	152	75	14	6	0.671 (2)	0.715
Girls	3	6	10	20	36	74	4	8		
Total	21	8	44	17	188	74	18	7		
Age										
9–11 years	10	17	8	63	37	7	4	7	12.171 (4)	0.016
12–14 years	2	10	6	30	12	60	0	0		
Above 14 years	9	5	30	16	139	72	14	7		
Total	21	8	44	16	188	69	18	7		

**Table 3:** Students' responses to perceived levels of knowledge on hand hygiene practices.

Students' perceived knowledge	Yes		No		*p* value
	*N*	%	*N*	%	

1.01. Is it important to wash your hands with soap when in school?	236	87.1	33	12.2	<0.001
1.02. Is it important to wash your hands with water to prevent diseases?	121	44.6	141	52.0	0.217
1.03. Is it important to wash your hands with soap to prevent diseases?	124	45.8	138	50.9	0.217
1.04. Is it important to wash your hands to remove germs?	74	27.3	187	69.0	0.387
1.05. Is it important to wash your hands to remove dirt?	108	39.9	153	56.5	<0.001
1.06. Is it important to wash your hands for personal hygiene?	224	82.7	37	13.7	<0.001
1.07. Have you ever been educated on how to wash your hands?	247	91.1	23	8.5	<0.001
1.08. Do you wash your hands before and after eating?	210	77.5	60	22.1	<0.001
1.09. Do you wash your hands after handling rubbish/garbage?	174	64.2	96	35.4	<0.001
1.10. Do you wash your hands before preparing food?	226	83.4	43	15.9	<0.001
1.11. Do you wash your hands after using the toilet?	141	52.0	128	47.2	<0.001
1.12. Do you wash your hands after playing with friends?	185	68.3	85	31.4	0.428
1.13. Do you wash your hands after coughing or blowing your nose?	185	68.3	85	31.4	<0.001
1.14. Did you wash your hands in school?	230	84.9	40	14.8	<0.001
1.15. Did you wash your hands in school with soap?	143	52.8	127	46.9	0.330
1.16. Is it necessary to dry your hands after washing?	207	76.4	63	23.2	<0.001

**Table 4:** Student responses on their hand hygiene performance.

Student performance	*N*	Percent	*p* value

How do you wash your hands?			<0.001
Wash hands with water	18	7	
Wash hands with water and soap	251	93	
Missing	2	1	
Total	271	100	
Where have you ever been educated on how to wash your hands?			<0.001
School	50	18	
Home	167	62	
Media	20	7	
Community	20	7	
Missing	14	5	
Total	271	100	
What is the best to use when washing your hands?			<0.001
Water only	7	3	
Water and soap	263	97	
Missing	1	0	
Total	271	100	
Which soap type is best to use in handwashing?			<0.001
Liquid soap	185	68	
Bar soap	46	17	
Powder detergent	5	2	
Do not know	32	12	
Missing	3	1	
Total	271	100	

**Table 5:** Distribution of student responses on their hand hygiene practice.

Students' perceived practice	Yes		No		*p* value
	*N*	%	*N*	%	

2.01. I always wash my hands before and after eating	238	88	28	10	<0.001
2.02. I always wash my hands with soap before and after eating	235	87	35	13	<0.001
2.03. I always wash my hands after visiting the toilet	232	86	31	11	<0.001
2.04. I always wash my hands with soap after handling garbage	239	88	31	11	<0.001
2.05. I always wash my hands after playing with friends	165	61	100	37	<0.001
2.06. I always wash my hands with soap after playing with friends	171	63	97	36	<0.001
2.07. I always wash my hands after blowing my nose or coughing	219	81	46	17	<0.001
2.08. I always wash my hands with water after blowing my nose or coughing	223	82	45	17	<0.001
2.09. I always wash my hands with soap after blowing my nose or coughing	223	82	45	17	<0.001
2.10. I always wash my hands when they are visibly dirty	235	87	28	10	<0.001
2.11. I always wash my hands with soap when they are visibly dirty	261	96	9	3	<0.001

**Table 6:** Bivariate analysis of students' means scores of hygiene practices according to their background.

Student background	*N* ^*∗*^	Mean	SD	Std. error mean	*T*-test^$^	*p* value

Gender						
Boy	218	10.05	2.333	0.158	1.510	0.132
Girl	53	9.51	2.259			
Age						
9–11 years	59	10.59	2.102	0.310	4.247	0.015
12–14 years	20	9.00	2.362			
Above 14 years	192	9.84	2.347			
Class level						
Elementary school	59	10.59	2.102	4.247	2	0.015
Secondary school	20	9.00	2.362			
High school	192	9.84	2.347			
Maternal education level						
No formal education	21	9.71	2.148	2.325	5	0.044
Elementary and secondary level	23	10.39	2.061			
High school level	69	10.35	1.984			
Undergraduate level	95	9.85	2.445			
Postgraduate level	37	8.89	2.706			
I do not know	7	10.57	1.512			
Paternal education level						
No formal education	15	10.87	1.407	1.214	5	0.303
Elementary and secondary level	14	10.14	2.070			
High school level	61	9.92	2.193			
Undergraduate level	74	9.66	2.495			
Postgraduate level	76	9.76	2.487			
I do not know	10	11.00	1.414			

^*∗*^The total number of complete cases is 271. ^$^F-score for more than two groups.

**Table 7:** Multivariate linear regression of student characteristics and means scores of hygiene practices.

Student characteristics	*N* ^*∗*^	Beta	*t*-value	*p* value	95% CI	

Gender						
Boy	218	1.00 (ref)				
Girl	53	−0.022	−0.305	0.761	−0.970	0.710
Age						
9–11 years	59	1.00 (ref)				
12–14 years	20	−0.174	−2.141	0.033^*∗*^	−2.963	−0.124
Above 14 years	192	−0.146	−2.125	0.035^*∗*^	−1.436	−0.055
Class level						
Elementary school	59	1.00 (ref)				
Secondary school	20	−0.140	−1.635	0.103	−2.739	0.254
High school	192	−0.128	−1.535	0.126	−1.487	0.184
Mother education						
No formal education	21	1.00 (ref)				
Elementary and secondary level	23	0.158	1.811	0.071	−0.115	2.753
High school level	69	0.312	2.468	0.014^*∗*^	0.336	2.989
Undergraduate level	95	0.223	1.691	0.092	−0.178	2.344
Postgraduate level	37	0.012	0.110	0.913	−1.321	1.477
I do not know	7	0.039	0.331	0.741	−2.835	3.979
Father education						
No formal education	15	1.00 (ref)				
Elementary and secondary level	14	−0.101	−1.210	0.228	−2.790	0.667
High school level	61	−0.242	−1.885	0.061	−2.751	0.060
Undergraduate level	74	−0.253	−1.866	0.063	−2.707	0.073
Postgraduate level	76	−0.166	−1.210	0.228	−2.250	0.538
I do not know	10	0.011	0.093	0.926	−2.758	3.033

^*∗*^The total number of complete cases is 271.

## Data Availability

Raw data are available from the corresponding author upon reasonable request.
